# Genomic characterization of *Salmonella* isolated from retail chicken and humans with diarrhea in Qingdao, China

**DOI:** 10.3389/fmicb.2023.1295769

**Published:** 2023-12-18

**Authors:** Wei Wang, Jing Cui, Feng Liu, Yujie Hu, Fengqin Li, Zhemin Zhou, Xiangyu Deng, Yinping Dong, Shaoting Li, Jing Xiao

**Affiliations:** ^1^NHC Key Laboratory of Food Safety Risk Assessment, China National Center for Food Safety Risk Assessment, Beijing, China; ^2^Qingdao Municipal Center for Disease Control and Prevention, Qingdao Institute of Preventive Medicine, Qingdao, China; ^3^Pharmaceutical Department, Qingdao Traditional Chinese Medicine Hospital (Qingdao Hiser Hospital) Qingdao Hiser Hospital Affiliated of Qingdao University, Qingdao, China; ^4^Key Laboratory of Alkene-carbon Fibres-based Technology and Application for Detection of Major Infectious Diseases, MOE Key Laboratory of Geriatric Diseases and Immunology, Pasteurien College, Suzhou Medical College, Soochow University, Suzhou, China; ^5^Center for Food Safety, University of Georgia, Griffin, GA, United States; ^6^Guangdong University of Technology, Guangzhou, China

**Keywords:** *Salmonella*, retail chicken, genome sequencing, humans with diarrhea, antimicrobial resistance

## Abstract

*Salmonella*, especially antimicrobial resistant strains, remains one of the leading causes of foodborne bacterial disease. Retail chicken is a major source of human salmonellosis. Here, we investigated the prevalence, antimicrobial resistance (AMR), and genomic characteristics of *Salmonella* in 88 out of 360 (24.4%) chilled chicken carcasses, together with 86 *Salmonella* from humans with diarrhea in Qingdao, China in 2020. The most common serotypes were Enteritidis and Typhimurium (including the serotype I 4,[5],12:i:-) among *Salmonella* from both chicken and humans. The sequence types were consistent with serotypes, with ST11, ST34 and ST19 the most dominantly identified. Resistance to nalidixic acid, ampicillin, tetracycline and chloramphenicol were the top four detected in *Salmonella* from both chicken and human sources. High multi-drug resistance (MDR) and resistance to third-generation cephalosporins resistance were found in *Salmonella* from chicken (53.4%) and humans (75.6%). In total, 149 of 174 (85.6%) *Salmonella* isolates could be categorized into 60 known SNP clusters, with 8 SNP clusters detected in both sources. Furthermore, high prevalence of plasmid replicons and prophages were observed among the studied isolates. A total of 79 antimicrobial resistant genes (ARGs) were found, with *aac(6′)-Iaa*, *bla*_TEM-1B_, *tet(A)*, *aph(6)-Id*, *aph(3″)-Ib*, *sul2*, *floR* and *qnrS1* being the dominant ARGs. Moreover, nine CTX-M-type ESBL genes and the genes *bla*_NMD-1_, *mcr-1.1*, and *mcr-9.1* were detected. The high incidence of MDR *Salmonella*, especially possessing lots of mobile genetic elements (MGEs) in this study posed a severe risk to food safety and public health, highlighting the importance of improving food hygiene measures to reduce the contamination and transmission of this bacterium. Overall, it is essential to continue monitoring the *Salmonella* serotypes, implement the necessary prevention and strategic control plans, and conduct an epidemiological surveillance system based on whole-genome sequencing.

## 1 Introduction

*Salmonella* is one of the common pathogens causing sporadic cases or outbreaks of gastroenteritis (Jain et al., 2020). In 2010, there were an estimated 153 million cases of non-typhoidal *Salmonella* enteric infections worldwide, of which about 50% were foodborne (Kirk et al., 2015; WHO, 2022). In the United States, foodborne salmonellosis causes an estimated 212,500 infections and 90 deaths annually (CDC, 2019). In China, *Salmonella* is the second most common bacteria causing foodborne outbreaks (Sun et al., 2021). In fact, consuming contaminated foods, especially poultry meat, directly threatens human health, which appears to be one of the major sources of human infection (Heredia and García, 2018; Pan et al., 2019; Yang et al., 2020). Poultry meat in the food production supply chain have been frequently associated with human salmonellosis cases and are an important cause of *Salmonella* transmission between poultry farms and humans (Antunes et al., 2016; Rincón-Gamboa et al., 2021). Furthermore, *Salmonella* is also a significant repository of antimicrobial resistance genes, posing substantial challenges to public health and security (Jajere, 2019).

Over recent years, resistance to cephalosporins have been increasingly reported in humans and poultry industry and the contribution of poultry products to the dissemination of extended-spectrum β-lactamase (ESBL) producing *Salmonella* strains, and the associated dangers for human health, is well documented in certain countries (Antunes et al., 2016). Of concern is the increased incidence of infections caused by ESBL-producing organisms, including *Salmonella*, because they are resistant not only to most of the β-lactam antimicrobials but also to other antimicrobial classes, leaving few treatment options and the potential for clinical outcomes worse than those of infections caused by non-ESBL-producing strains (Wei et al., 2019; Wang et al., 2020a). Meanwhile, carbapenems and colistin are often used as the last-line treatment for infections caused by multidrug-resistant (MDR), especially ESBL-producing gram-negative bacteria. However, selective pressure from the overuse or misuse of both antimicrobials has resulted in the emergence of carbapenem- and colistin-resistant Enterobacteriaceae (CRE) (Nang et al., 2019; Octavia et al., 2020). Regarding *Salmonella* isolates, recent studies have demonstrated their resistance to these critical antimicrobial drugs (Wang et al., 2017; Fan et al., 2022; Zhang et al., 2022b).

The New Delhi metallo (NDM)-β-lactamases that can hydrolyze almost all β-lactam antibiotics, have become one of the most commonly reported carbapenemase resistance mechanisms worldwide (Poirel et al., 2010). According to the Comprehensive Antibiotic Resistance Database (CARD^1^), 43 variants of NDM genes have been identified in 2022, with NDM-1 and NDM-5 being particularly widespread among *Enterobacteriaceae* including *Salmonella* (Iovleva and Doi, 2017). Colistin resistance in bacteria has become another significant threat to food safety and public health, and its development was mainly attributed to the plasmid-mediated *mcr* genes. Currently, a variety of *mcr* genes, including *mcr-1* to *-10* have been reported with *mcr-9.1* being prevalent in *Salmonella* (Ling et al., 2020; Hussein et al., 2021). Hence, the increasing antimicrobial resistance in *Salmonella* needs to be monitored.

Our previous investigation has described the emergence of the *bla*_NDM-1_ gene in *Salmonella* recovered from chicken carcass in slaughterhouse, while the *mcr* genes were also detected from living chicken in Qingdao (Wang et al., 2017; Zou et al., 2021). However, there is limited available information on the surveillance and genomics studies of *Salmonella* in retail chicken in Qingdao. In the current study, we therefore investigated the prevalence, AMR, and genomic characteristics of *Salmonella* from chilled chicken carcasses and humans with diarrhea in Qingdao. Specially, we identified four *Salmonella* isolates carrying the plasmid-borne *mcr-1.1*, *mcr-9.1*, and NDM-1 genes in this study.

## 2 Materials and methods

### 2.1 Bacterial isolation

A total of 360 chilled chicken carcasses were collected from local markets in Qingdao, China in 2020. All samples were subjected to qualitative analysis for *Salmonella* using an enrichment method described by the National Food Safety Standard of China-Food microbiological examination, *Salmonella* (GB 4789.4–2016). Finally, presumptive *Salmonella* was selected for biochemical confirmation using API 20E test identification test strips (bioMérieux, Marcy l′ Etoile, France), as well as for molecular identification using PCR assay targeting the *invA* gene (Ed-Dra et al., 2018). In addition, 86 *Salmonella* isolates from humans with diarrhea collected from enteric clinic settings in the study year in Qingdao, were also included in the study (Supplementary Table S1). The isolates were serotyped using O- and H-antigens by slide agglutination with hyperimmune sera, and the serotypes of *Salmonella* samples were identified following the Kauffmann-White scheme (Grimont and Weill, 2007). All confirmed *Salmonella* isolates were stored in brain heart infusion broth with 40% glycerol (Land Bridge, Beijing, China) at −80°C. One isolate was retained from each sample. All the procedures performed in studies involving human participants were in accordance with the ethical standards of the Research Ethics Committee of China National Center of Food Safety Risk Assessment, Beijing, China (approval no. 2014003).

### 2.2 Antimicrobial susceptibility testing

Antimicrobial susceptibility testing (AST) of the *Salmonella* isolates was evaluated using the broth dilution method by the Biofosun Gram-negative panels (Shanghai Biofosun Biotech, China) by the manufacturer’s instructions. The MICs of 13 antimicrobial agents, including ampicillin (AMP), ceftazidime (CAZ), cefotaxime (CTX), cefoxitin (CFX), imipenem (IPM), meropenem (MEM), trimethoprim-sulfamethoxazole (SXT), gentamicin (GEN), tetracycline (TET), ciprofloxacin (CIP), nalidixic acid (NAL), chloramphenicol (CHL), and polymyxin E (CT) were determined, and the results were interpreted using Clinical and Laboratory Standards Institute guidelines (CLSI, 2017). *Escherichia coli* ATCC 25922 was used as quality control. Isolates showed resistance to all tested antimicrobials in this study were defined as pan-resistance, while isolates showed resistance to at least three classes of antimicrobials were defined as MDR.

### 2.3 Whole-genome sequencing and *in silico* analysis

Whole-genome sequencing of the *Salmonella* isolates from both chicken and humans was carried out using the Illumina NovaSeq PE150 at the Beijing Novogene Bioinformatics Technology Co., Ltd. Trimmomatic (Bolger et al., 2014), FastQC,^2^ SPAdes v3.14 (Bankevich et al., 2012), and Prokka v1.14.5 (Seemann, 2014) were used for reads quality control, assembly, and annotation. MLST v2.19.0^3^ was used for identifying the sequence type (ST). SeqSero2 v1.2.1^4^ was used for identifying the *Salmonella* serotypes (Zhang et al., 2019). Antimicrobial resistance genes (ARGs) were identified using ResFinder v4.0 (Bortolaia et al., 2020). Virulence genes were identified by ABRicate v1.01^5^ using the VFDB database (Liu et al., 2022) with 80% identity and 80% query coverage cutoffs. Plasmid replicon types were identified using PlasmidFinder v2.1 (Carattoli and Hasman, 2020). Prophage predictions were carried out using PHASTER to explore intact prophages (Arndt et al., 2016). Pan-genome analysis was performed using Roary v3.13.0 (Page et al., 2015). Core genome phylogeny was built using PhyML v3.3.20200621 (Guindon et al., 2010). The gene presence/absence of ARGs, virulence genes, plasmid replication types, and prophage types, along with the phylogenetic tree were visualized through R script (v3.6.2) with the package ggtree (Yu, 2020). The genomes were submitted to EnteroBase for cgMLST profiling (Zhou et al., 2020) and submitted to NCBI Pathogen Detection portal for SNP clustering (Timme et al., 2020).

### 2.4 Statistical analysis

The Chi-square test was performed to analyze differences between groups. Data analysis was performed using SPSS 20.0 (SPSS, Chicago, United States). All statistical tests were two-sided; *p* < 0.05 were considered statistically significant.

### 2.5 Data availability statement

The sequences obtained in this study have been deposited in the NGDC Genome Sequence Archive^6^ under accession number CRA012442.

## 3 Results

### 3.1 Distribution of *Salmonella* serotypes and sequence types in retail chilled chicken carcasses and humans with diarrhea

**Figure 1:**
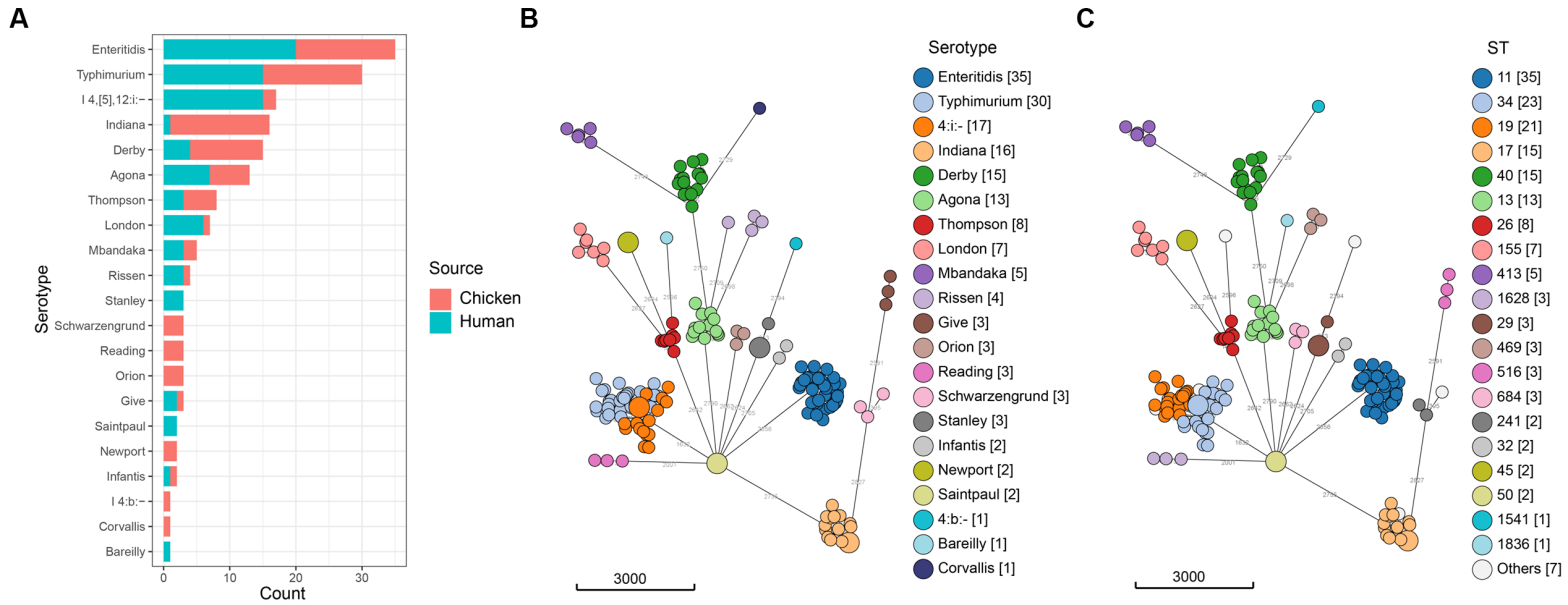
Distribution of *Salmonella* isolates. **(A)** Number of isolates from retail chilled chicken carcasses and humans with diarrhea. **(B)** Minimum spanning tree of the 174 *Salmonella* genomes colored by serotype. **(C)** Minimum spanning tree of the 174 *Salmonella* genomes colored by ST. The minimum spanning tree was built using cgMLST allelic profiles with the MSTreeV2 algorithm.

A total of 88 (24.4%) *Salmonella* isolates were recovered from 360 retail chilled chicken carcasses in the local markets in Qingdao, China in 2020. Using both traditional and *in silico* serotyping methods, which yielded identical results, we determined that the 88 isolates represented 18 different serotypes (Figure 1, Supplementary Table S1). The serotypes of Enteritidis, Indiana, and Typhimurium were frequently detected (each was 17.0%, 15/88) among *Salmonella* from retail chilled chicken carcasses, followed by Derby (12.5%, 11/88), Agona (6.8%, 6/88), and Thompson (5.7%, 5/88) (Supplementary Table S2). The results also demonstrated that the 86 isolates collected from humans with diarrhea belonged to 15 different serotypes, with Enteritidis (23.3%, 20/86), Typhimurium (17.4%, 15/86), and I 4,[5],12:i:- (17.4%, 15/86) frequently detected, followed by Agona (8.1%, 7/86), London (7.0%, 6/86), and Derby (4.7%, 4/86) (Supplementary Table S2). There were six and three serotypes were isolated only in retail chilled chicken carcasses or humans with diarrhea, respectively.

The sequence types of all 174 studied *Salmonella* isolates showed strong clustering by serotypes (Figure 1, Supplementary Table S3). The most prevalent sequence type was ST11 (20.1%, 35/174, *S.* Enteritidis), followed by ST34 (13.2%, 23/174, *S.* Typhimurium/*S*. I 4,[5],12:i:-), ST19 (12.1%, 21/174, *S.* Typhimurium). The ST17 (8.6%, 15/174), ST40 (8.6%, 15/174), and ST13 (7.5%, 13/174) clones were detected in *S.* Indiana, *S*. Derby, and *S*. Agona, respectively. We also noticed that each serotype was associated with one ST, except for *S.* Typhimurium (ST19, ST34, ST36, ST99, and ST3557), *S.* Indiana (ST17 and ST3558), *S*. Rissen (ST469 and ST1836), and *S*. Schwarzengrund (ST96 and ST241).

### 3.2 Antimicrobial resistance phenotype

**Figure 2:**
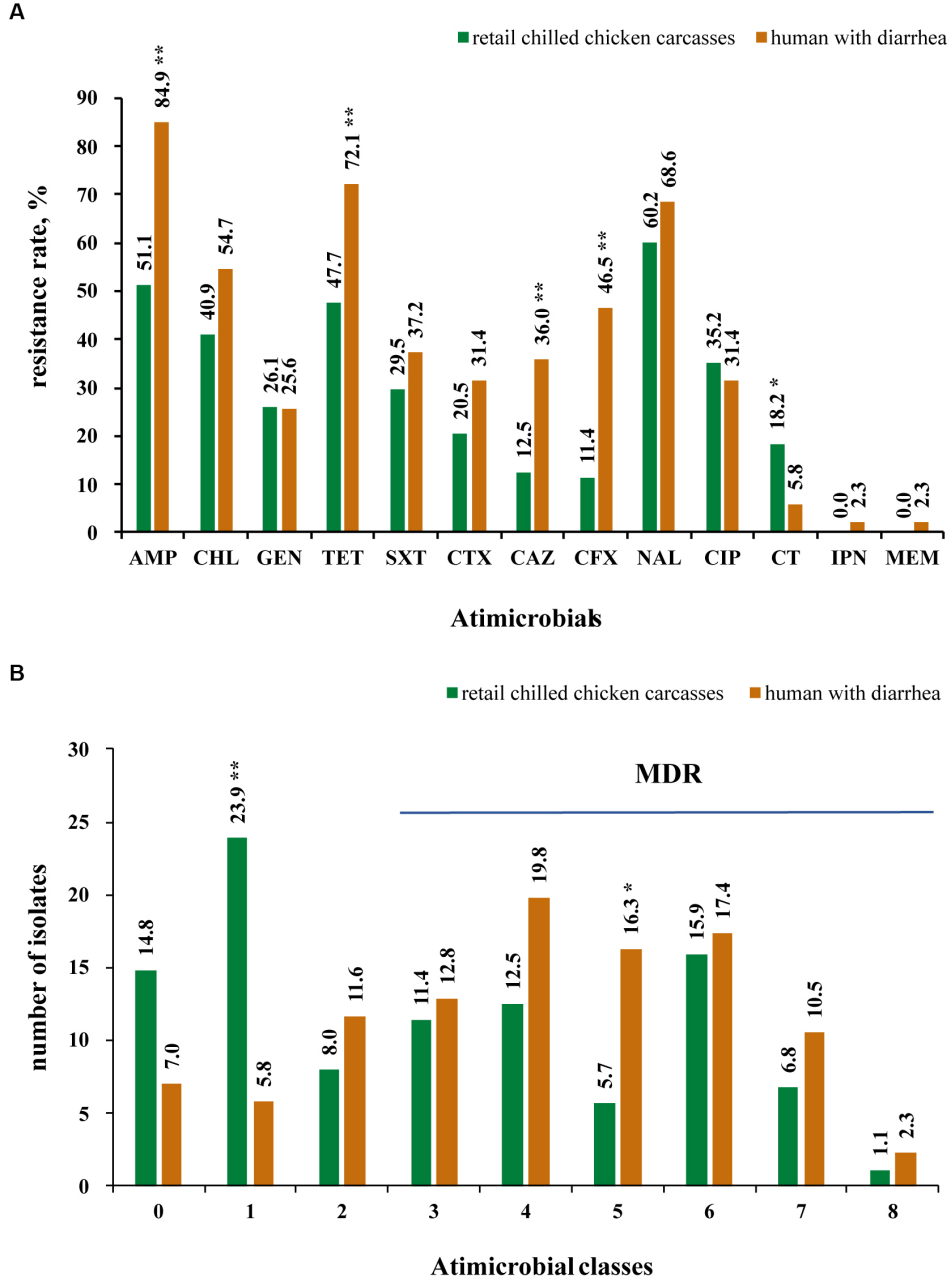
Antimicrobial resistance of 174 Salmonella isolates collected from retail chilled chicken carcasses and humans with diarrhea in this study. **(A)** Antimicrobial resistance rate of 174 Salmonella isolates to 13 antimicrobials. **(B)** Number of Salmonella isolates showing resistance to studied antimicrobials. * and ** mean significant difference with the *p* value less than 0.05 and 0.01, respectively.

The results showed that 85.2% (75/88) of the *Salmonella* from retail chilled chicken carcasses were resistant to at least one antimicrobial agent, whereas 13 isolates (14.8%) were susceptible to all tested antimicrobials (Supplementary Table S1). In detail, the top four resistant antimicrobials were nalidixic acid (60.2%, 53/88), ampicillin (51.1%, 45/88), tetracycline (47.7%, 42/88) and chloramphenicol (40.9%, 36/88) (Figure 2A). Meanwhile, 47 isolates (53.4%) were MDR (i.e., could resist ≥3 antimicrobial classes) (Figure 2B). A total of 23 isolates (26.1%) were resistant to at least one of the third-generation cephalosporins (ceftazidime and cefotaxime), including 13 *S.* Indiana (86.7%, 13/15), 5 *S*. Thompson (5/5), 2 *S.* Typhimurium (13.3%, 2/15), 1 *S*. Agona (1/6), 1 *S*. Rissen (1/1), and 1 *S.* Enteritidis (6.7%, 1/15) (Supplementary Table S1). Most of these 23 isolates showed concurrent resistance to ciprofloxacin (*n* = 17). Notably, Polymyxin E resistance was observed in 16 isolates, including 8 *S.* Enteritidis, 2 *S*. Derby, and one each of *S*. Agona, *S*. I 4,[5],12:i:-, *S.* Indiana, *S*. Reading, *S*. Schwarzengrund, and *S.* Typhimurium.

Among the *Salmonella* isolates from humans with diarrhea, 93.0% (80/86) displayed resistance to at least one antimicrobial agent, while the remaining 6 isolates (7.0%) were susceptible to all tested antimicrobials (Supplementary Table S1). In detail, the top four resistant antimicrobials were also found to be ampicillin (84.9%, 73/86), tetracycline (72.1%, 62/86), nalidixic acid (68.6%, 59/86), and chloramphenicol (54.7%, 47/86), while 65 isolates (75.6%) were MDR (Figures 2A,B). A total of 34 isolates (39.5%) were resistant to at least one of the third-generation cephalosporins (ceftazidime and cefotaxime), including 9 *S.* Enteritidis (45%, 9/20), 8 *S.* Typhimurium (50%, 8/16), 7 *S*. I 4,[5],12:i:- (50%, 7/14), 2 *S*. Derby (2/4), 2 *S*. Saintpaul (2/2), 2 *S*. Stanley (2/3), one of each *S*. Agona (1/7), *S*. Bareilly (1/1), *S.* Infantis (1/1), *S*. Mbandaka (1/3), and *S*. Thompson (1/3), while 13 of these 34 isolates showed concurrent resistance to ciprofloxacin (Supplementary Table S1). Five isolates including 4 *S.* Enteritidis and 1 *S*. Agona showed resistant to polymyxin E. Moreover, two isolates (one of each *S.* Enteritidis and *S.* Infantis) were found to be resistant to carbapenems (imipenem and meropenem).

Furthermore, the *Salmonella* isolates from humans with diarrhea displayed higher resistance rates to several antimicrobials, including ampicillin, tetracycline, ceftazidime, and cefotaxime (*p* < 0.01), than the isolates from retail chilled chicken carcasses (Figure 2A). In addition, the human-associated *Salmonella* isolates showed higher MDR (*p* < 0.05) than those from retail chilled chicken carcasses (Figure 2B).

The AMR of the mainly detected *Salmonella* serotypes (*n* > 10) from retail chilled chicken carcasses and humans with diarrhea were estimated that the *S.* Indiana isolates exhibited the most pan-resistance (100%) and MDR (93.8%), followed by *S*. I 4,[5],12:i:- (pan-resistance, 100%; MDR, 87.5%), and *S.* Enteritidis (pan-resistance, 97.1%; MDR, 71.4%) (Table 1). Of each serotype, the human-associated isolates *S*. Agona (71.4%), *S*. I 4,[5],12:i:- (92.9%), and *S.* Typhimurium (81.3%) showed higher MDR than those of chicken-associated isolates (16.7, 50, and 40%, respectively).

**Table 1:** AMR of the mainly detected *Salmonella* serotypes, %(n/N).

Serotypes	Total		Retail chilled chicken carcasses		Humans with diarrhea	
	Pan-resistant	MDR	Pan-resistant	MDR	Pan-resistant	MDR
Agona	84.6 (11/13)	46.2 (6/13)	83.3 (5/6)	16.7 (1/6)	85.7 (6/7)	71.4 (5/7)^*^
Derby	93.3 (14/15)	60 (9/15)	90.9 (10/11)	45.5 (5/11)	100 (4/4)	100 (4/4)
Enteritidis	97.2 (33/35)	72.2 (25/35)	100 (15/15)	60 (9/15)	95 (19/20)	80 (16/20)
I 4,[5],12:i:-	100 (16/16)	87.5 (14/16)	100 (2/2)	50 (1/2)	100 (14/14)	92.9 (13/14)^*^
Indiana	100 (16/16)	93.8 (15/16)	100 (15/15)	93.3 (14/15)	100 (1/1)	100 (1/1)
Typhimurium	83.9 (26/31)	61.3 (19/31)	80 (12/15)	40 (6/15)	87.5 (14/16)	81.3 (13/16)^*^

* means significant difference with the *p* value less than 0.05.

### 3.3 Phylogenetic analysis revealed genomic diversity of *Salmonella*

**Figure 3:**
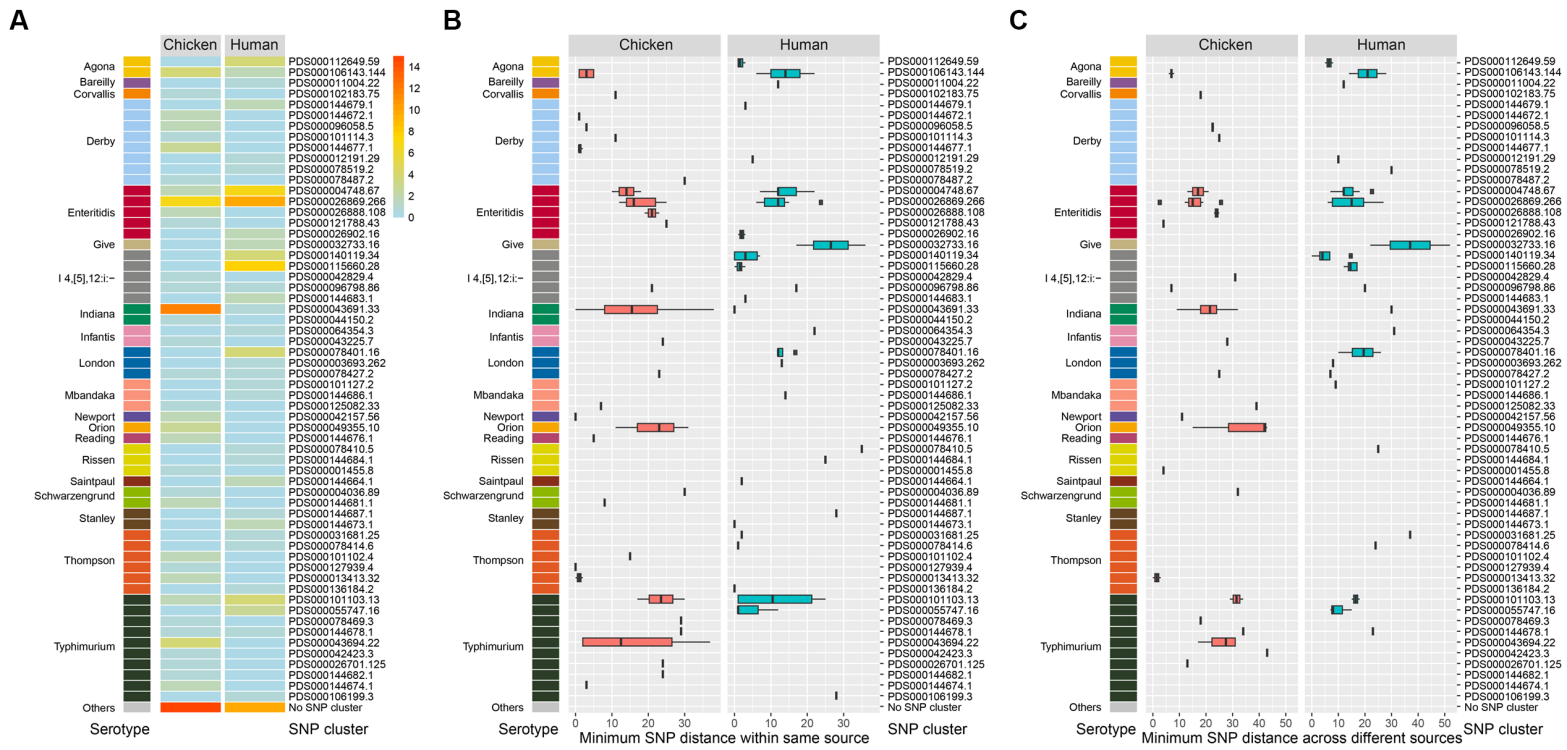
Genomic diversity of *Salmonella* isolates. **(A)** Heat map of serotype distribution and the designated SNP clusters from different sources. Color indicates the number of isolates. **(B)** Box plot of minimum SNP distance within same source of the designated SNP clusters of different serotypes. **(C)** Box plot of minimum SNP distance across different sources of the designated SNP clusters of different serotypes.

All of the 174 isolates including 88 chicken-associated and 86 human-associated *Salmonella* isolates were subjected to whole genome sequencing (WGS). Based on data from the NCBI Pathogen Detection, 149 out of the 174 isolates (85.6%) could be categorized into known SNP clusters (*n* = 60). The distribution of SNP clusters differed between retail chicken and humans, with 27 unique SNP clusters found in chicken source and 25 unique cluster found in human source (Figure 3). Eight SNP clusters (PDS000078427.2, PDS000043691.33, PDS000096798.86, PDS000106143.144, PDS000144678.1, PDS000004748.67, PDS000101103.13, and PDS000026869.266) were detected in both sources, which belonged to six serotypes (*S*. London, *S.* Indiana, *S*. I 4,[5],12:i:-, *S*. Agona, *S.* Typhimurium, and *S.* Enteritidis) (Supplementary Table S4), revealing diverse origins of *Salmonella* isolated in this study.

Notably, the top six prevalent SNP clusters among the 174 isolates included PDS000026869.266, PDS000043691.33, PDS000004748.67, PDS000115660.28, PDS000101103.13, and PDS000106143.144. All of these, except PDS000115660.28, were detected in both chicken and humans. PDS000115660.28 only contained isolates from humans. The most prevalent SNP cluster PDS000026869.266 contained 10 isolates from humans and 7 isolates from retail chicken.

*S.* Typhimurium/*S*. I 4,[5],12:i:- displayed the highest diversity with 15 detected SNP clusters, followed by *S*. Derby with 8, *S*. Thompson with 6, and *S.* Enteritidis with 5 (Figure 3A). Among the designated SNP clusters (*n* = 35) of the chicken isolates, 21 of them had a minimum SNP distance within environmental source below 21 (Figure 3B), while 14 of them had a minimum SNP distance within human source below 21 (Figure 3C). In contrast, 24 of the designated SNP clusters (*n* = 33) of the human-associated isolates had a minimum SNP distance within human source below 21 (Figure 3B), while 15 clusters had a minimum SNP distance within environmental source below 21 (Figure 3C). These findings demonstrated that a large number of the designated SNP clusters contained isolates circulating in both human and environmental sources (Pightling et al., 2018). For the 25 isolates that did not fit into designated SNP clusters, 15 were from retail chicken and 10 were from humans. The most common serotypes among these were *S.* Typhimurium, *S.* Enteritidis, and *S*. Agona.

### 3.4 MGE profiles

**Figure 4:**
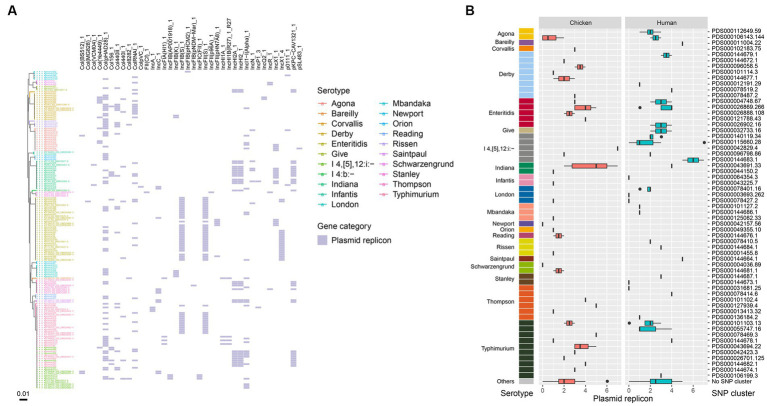
Plasmid replicons in the *Salmonella* genomes. **(A)** Core genome phylogeny of the *Salmonella* genomes and distribution of plasmid replicons. Color of the heatmap indicates the presence of plasmid replicons. **(B)** Box plot of plasmid replicon counts of the designated SNP clusters of different serotypes.

Plasmid replicons were detected in a majority of the bacterial isolates from this study, with only 9.8% (17/174) showing no evidence of plasmids in their genomes. Ten of the human-associated isolates and seven of the chicken-associated isolates had no detected plasmid replicons. A total of 38 plasmid replicons were identified among the 174 isolates, with 5 isolates showing the highest number of 7 plasmid replicons (Figure 4A). The most frequently identified plasmid replicons were Col(pHAD28), IncFIB(S), and IncFII(S) (Figure 4A). Conversely, less common types such as Col(MG828), ColpVC, FII(*Cf*), IncFIB(pHCM2), IncFIB(pNDM-Mar), IncFII(p96A), and pSL483 were present in certain single isolate, respectively. Additionally, the prevalence of plasmid replicons exhibited variations over isolates from retail chicken and humans. For instance, strains isolated in chicken showed a higher prevalence of IncHI2A, IncHI2 and pKPC-CAV1321, while those isolated in humans showed a higher prevalence of IncI1-I(Alpha) and Col440II. When considering different serotypes, *S.* Typhimurium and *S.* Indiana exhibited the largest number of plasmid types (16 in total, respectively), followed by *S*. I 4,[5],12:i:- (15 in total), and *S.* Enteritidis (14 in total) (Figure 4A). Notably, isolates from *S.* Enteritidis were more likely to carry plasmid replicons of IncFIB(S), IncFII(S), and IncX1, isolates from *S.* Typhimurium were more likely to carry Col(pHAD28), IncFIB(S), and IncFII(S), and isolates from *S.* Indiana were more likely to carry IncHI2A, IncHI2, and pKPC-CAV1321 (Figure 4A). Among the designated SNP clusters, PDS000043691.33 (*S.* Indiana), PDS000115660.28 (*S*. I 4,[5],12:i:-), PDS000042829.4 (*S*. I 4,[5],12:i:-), and PDS000144683.1 (*S*. I 4,[5],12:i:-) each harbored the highest number of plasmid replicon types, with seven types each (Figure 4B). Chicken-associated SNP clusters had a higher median number of plasmid replicons (3) compared to human-associated SNP clusters (2) (Figure 4B).

**Figure 5:**
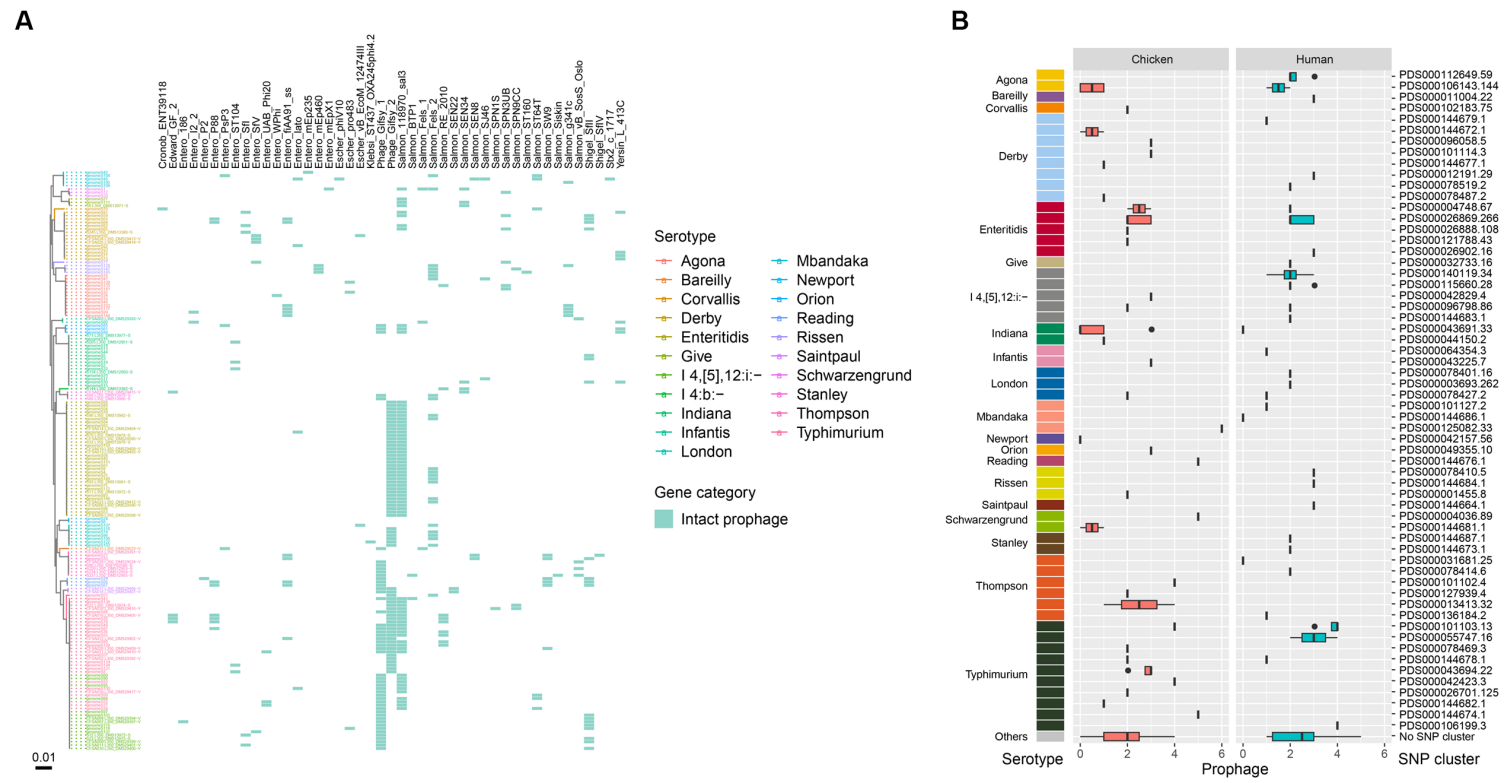
Prophages in the *Salmonella* genomes. **(A)** Core genome phylogeny of the *Salmonella* genomes and distribution of intact prophages. Color of the heatmap indicates the presence of prophages. **(B)** Box plot of prophage counts of the designated SNP clusters of different serotypes.

We also observed a high prevalence of intact prophages among the isolates, with only 17 lacking any. A total of 45 intact prophages were identified among the 174 isolates, with 31 isolates harboring only one prophage type (Figure 5A). Most isolates (72.4%, 126/174) contained at least two intact prophages. Seven isolates harbored more than 5 intact prophages, which belonged to serotypes such as Mbandaka, Typhimurium, Schwarzengrund, and Reading. Notably, 64 isolates, representing various serotypes, contained both Gifsy (Gifsy-1/Gifsy-2) and Salmon 118,970 sal3 prophages (Figure 5A). Among the various prophages identified, Gifsy-2 was more commonly present in isolates from humans (41/86) than retail chicken (26/88). When examining different serotypes, the Gifsy and Salmon 118,970 sal3 prophages were more likely to be carried by *S.* Enteritidis, *S.* Typhimurium, and *S*. I 4,[5],12:i:-, and were less frequently found in other serotypes. All the *S.* Enteritidis isolates carried Gifsy-2 and Salmon 118,970 sal3 prophages (Figure 5A). It is worth noting that all the isolates of *S*. I 4,[5],12:i:- carried Gifsy-2 other than Gifsy-1 prophage, while isolates of *S.* Typhimurium carried both types of Gifsy-1 and Gifsy-2 prophages. Most *S.* Indiana isolates (9/16) did not carry any intact prophages, while a small portion of *S.* Indiana isolates (6/16) carried only one intact prophage. The two *S.* Newport isolates were found to be free of intact prophages. In addition, SNP clusters that were associated a high number of intact prophage types (more than 5) contained PDS000144674.1 of *S.* Typhimurium (2/30), PDS000144676.1 of *S*. Reading (2/3), PDS000004036.89 of *S*. Schwarzengrund (1/3), and PDS000125082.33 of *S*. Mbandaka (1/5) (Figure 5B).

### 3.5 ARG profiles

**Figure 6:**
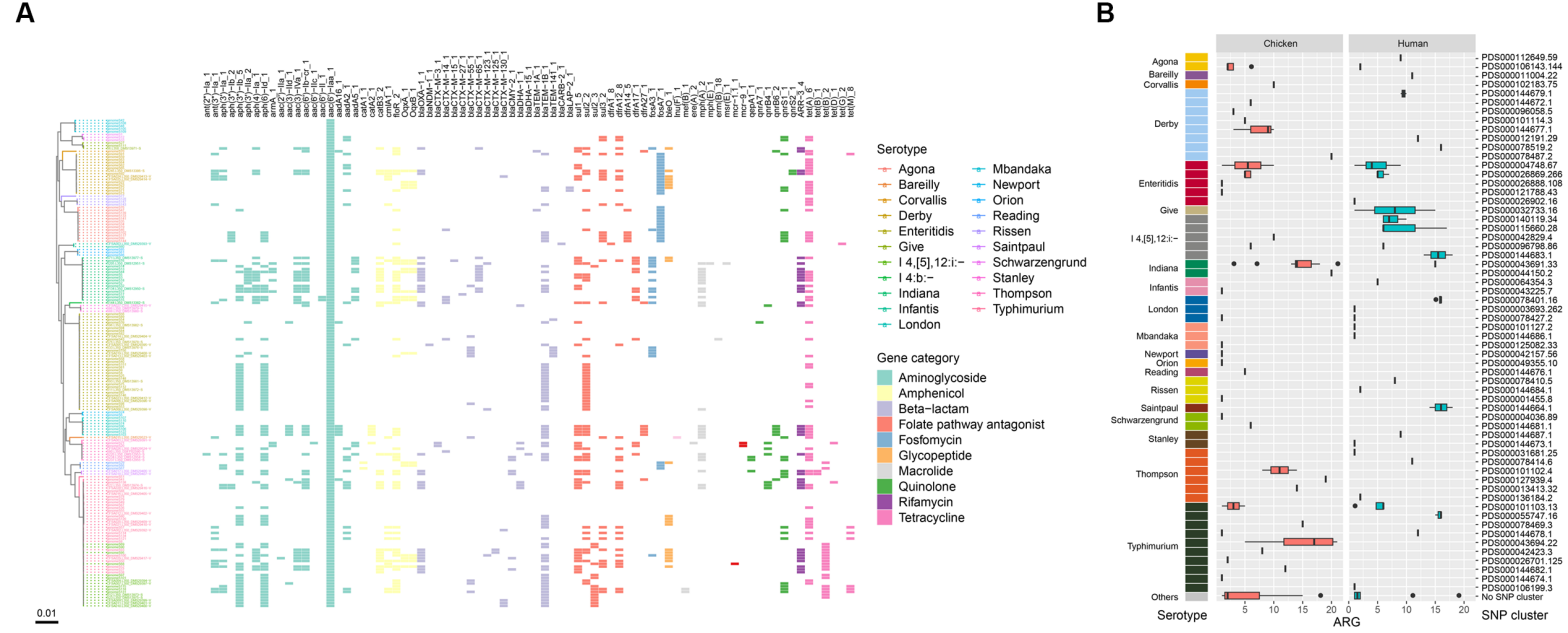
ARGs in the *Salmonella* genomes. **(A)** Core genome phylogeny of the *Salmonella* genomes and distribution of ARGs. Color of the heatmap indicates the presence of ARGs. **(B)** Box plot of ARG counts of the designated SNP clusters of different serotypes.

To determine the presence of resistance genes, all 174 genome sequences were screened against the ResFinder database. A total of 79 different antibiotic resistance genes (ARGs) were identified in the isolates (Figure 6A). The majority of these ARGs belonged to beta-lactam resistance (*n* = 19) and aminoglycoside resistance (*n* = 19) categories. Among the CTX-M-type beta-lactamases, 9 different *bla*_CTX-M_ genes were detected, with *bla*_CTX-M-55_ (*n* = 11), *bla*_CTX-M-65_ (*n* = 10), and *bla*_CTX-M-14_ (*n* = 6) being the most prevalent (Figure 6A).

Notably, all isolates were positive for the aminoglycoside resistance gene *aac(6′)-Iaa*, and a large number of them carried the beta-lactam resistance gene *bla*_TEM-1B_ (*n* = 67), the tetracycline resistance gene *tet(A)* (*n* = 64), aminoglycoside resistance genes *aph(6)-Id* (*n* = 65) and *aph(3″)-Ib* (*n* = 60), the sulfonamide resistance gene *sul2* (*n* = 50), and the amphenicol resistance gene *floR* (*n* = 50) (Figure 6A). Six types of quinolone resistance genes were found, including *qnrS1* (*n* = 23), *qnrB4* (*n* = 7), *qnrB6* (*n* = 6), *qepA1* (*n* = 3), *qnrS2* (*n* = 2), and *qnrA7* (*n* = 1). Additionally, one *S.* Typhimurium isolate was positive for the colistin resistance gene *mcr-1.1*, and two *S.* Thompson isolates carried *mcr-9.1* (Figure 6A). All these *mcr*-positive isolates were from chicken. One *S.* Enteritidis isolate was positive for the *bla*_NDM-1_ gene. Furthermore, the *mcr-1.1* gene was detected on an IncHI2 plasmid replicon, while the *bla*_NDM-1_ gene was carried on an IncC plasmid. The plasmid carrying the *mcr-9.1* gene could not be identified as the gene was located on small contigs with a length of ~2,500 bp (data not shown).

Due to the high prevalence of genes related to aminoglycoside and beta-lactam resistance, all isolates contained at least one ARG in their genomes (Figure 6A). Three isolates, genomeS31, genomeS44, and genomeS52, harbored the largest number of ARGs (*n* = 21), which belonged to two serotypes, *S.* Indiana and *S.* Typhimurium. When considering different serotypes, *S.* Indiana exhibited the largest number of ARGs (14.5 in median), followed by *S*. Thompson (12.5 in median), *S*. I 4,[5],12:i:- (6 in median), and *S.* Typhimurium (5.5 in median). SNP clusters that were associated with higher number of ARGs included PDS000043691.33 (14 in median, *S.* Indiana), PDS000044150.2 (20, *S.* Indiana), PDS000055747.16 (16 in median, *S.* Typhimurium), PDS000043694.22 (17 in median, *S.* Typhimurium), PDS000144683.1 (15.5 in median, *S*. I 4,[5],12:i:-), PDS000127939.4 (19, *S*. Thompson), PDS000078401.16 (16 in median, *S*. London), PDS000144664.1 (16 in median, *S*. Saintpaul), and PDS000078519.2 (16, *S*. Derby) (Figure 6B).

It is worth noting that most of the ARGs were detected on plasmid sequences (Supplementary Figure S1A). The aminoglycoside resistance gene *aac(6′)-Iaa* presented in all the isolates was found located on chromosome sequences (Supplementary Figure S1B). Additionally, a total of 40 isolates were found carrying no ARGs other than the *aac(6′)-Iaa* gene, which belonged to a variety of serotypes such as *S.* Typhimurium (*n* = 10), *S.* Enteritidis (*n* = 9), *S*. Mbandaka (*n* = 5), etc.

## 4 Discussion

*Salmonella* is one of the most widespread pathogenic foodborne bacteria in Chinese food products and retail chicken have been recognized as one of the major sources of human salmonellosis (Paudyal et al., 2018). Moreover, there has been an increase in reports of the dissemination of resistant *Salmonella* from chicken to humans through the chicken production chain (Kongsanan et al., 2021; Manzari et al., 2022). In this study, we surveyed the prevalence, serovar distribution, MDR, and genomic characteristics of *Salmonella* in chilled chicken carcass and humans with diarrhea sources in Qingdao, China in 2020.

Overall, 24.4% of the retail chilled chicken carcass samples purchased in 2020 in local markets contained *Salmonella*. This prevalence is lower than what was found in other provinces (average, 39.3%) in China (Wang et al., 2020a), Brazil (31.5%) (Perin et al., 2020), but higher than those in Korea (14.1%) (La et al., 2021), the USA (17.9%) (Peng et al., 2016), and European Union (7.1%) (Gonçalves-Tenório et al., 2018). Thus, chicken meat in retail may be an important source of human salmonellosis in China. Besides, data reported that *Salmonella* prevalence in chilled poultry meat was statistically higher than that of frozen poultry meat and ambient poultry meat in China (Sun et al., 2021). In China, immersion chilling is employed more frequently and Chinese consumers have a preference for chilled meat (25% market share) over frozen meat (15% market share) (Liu et al., 2017). However, once a sample is contaminated with *Salmonella* during the immersion process, the contamination may spread among the whole batch of carcasses, leading to an increase in the prevalence of pathogens on finished products (Marmion et al., 2021). Therefore, in response to potential public health pressures, more effective intervention strategies during immersion chilling should be implemented to control the quality and safety of chicken products.

Serotype results suggested a wide range of *Salmonella* serotypes present in retail chilled chicken carcasses and humans with diarrhea. The dominance of *S.* Enteritidis and *S.* Typhimurium among the studied *Salmonella* isolates, is consistent with the data obtained from chicken and human previously in China and other regions worldwide (EFSA and ECDC, 2018; Balasubramanian et al., 2019; Wang et al., 2020a). Besides, *S.* Indiana is another frequently detected serotype among chicken samples and is also detected from humans with diarrhea in this study. Accordingly, since 2009, the presence of *S.* Indiana has seen a remarkable increase, which has become one of the top three common serotypes in China (Zhang et al., 2022a). *S.* Indiana has been isolated from a wide and diverse variety of sample sources in 18 provinces throughout China and was most frequently recovered from food, followed by animals, the environment and then people, especially children under 6 years old with low immunity (Gong et al., 2017). What is more, *Salmonella* serovars Enteritidis, Typhimurium, and Indiana are also reported as the most common serotypes associated with human infections and outbreaks (Kuang et al., 2018b; Wu et al., 2018). Thus, the high prevalence of these *Salmonella* serotypes in chicken carcasses and humans with diarrhea indicates a significant risk to public health. The monitoring of the emergence and prevalence of these *Salmonella* serotypes is essential for the better control of salmonellosis.

The combination of MLST and serotype detection can facilitate research on the hereditary and evolutionary relationships of *Salmonella* (Xu et al., 2022). The most common *Salmonella* sequence types were found to be ST11, ST34, and ST19 in this study, where the corresponding serotypes were *S.* Enteritidis and *S.* Typhimurium (including *S*. I 4,[5],12:i:-). This is consistent with findings from previous reports in China (Wang et al., 2020b; Xu et al., 2022). Accordingly, among *S.* Enteritidis worldwide, ST11 is the predominant sequence type, accounting for 89% of the sequence types in the EnteroBase database (Ashton et al., 2016). However, a recent report from Malaysia showed that ST1925 was dominantly detected among chicken-associated *S.* Enteritidis strains (Zakaria et al., 2022). In America, the major prevalent STs are ST34, ST33, and ST11 (Pethplerdprao et al., 2017), which shows that STs may be associated with regions. Furthermore, the other mainly identified sequence types, ST13 (*S*. Agona), ST17 (*S.* Indiana), and ST40 (*S*. Derby), were also previously reported to be associated with salmonellosis in human (Luo et al., 2022; Sun et al., 2022; De Jesus Bertani et al., 2023). Observations from the present study therefore provided important evidence and confirmed further that these types of *Salmonella* can serve an important role in human diarrhea in Qingdao, China.

Herein, we found that 53.4 and 75.6% of retail chilled chicken carcass- and humans with diarrhea-associated *Salmonella* isolates were MDR, respectively. High percentages of MDR strains were also observed in *Salmonella* isolated from poultry (Li et al., 2022), food animals (Tang et al., 2023) and humans (Chen et al., 2022) in various regions of China. The surge in antimicrobial-resistant *Salmonella* isolates is recognized as a crucial public health issue (Eng et al., 2015). The major of *Salmonella* isolates among both sources were resistant to four antimicrobials of nalidixic acid, ampicillin, tetracycline and chloramphenicol, which are the first-line drugs used against bacterial infection in animal farms worldwide (Nhung et al., 2016; Lekagul et al., 2019). This finding is consistent with the literature from different countries, including China (Tamang et al., 2011; Cai et al., 2016; EFSA and ECDC, 2018). Our results also suggested that *Salmonella* isolates in this study showed high level resistance to third generation cephalosporins (ceftazidime and cefotaxime) and quinolone (ciprofloxacin), which are the first-line drugs used against bacterial infection in clinics (Tack et al., 2020). These findings are regarded as significant threats to public health, leading to limited drugs of choice for salmonellosis infection treatment in animals and humans. The high AMR rate of *S.* Enteritidis, *S.* Typhimurium (including *S*. I 4,[5],12:i:-) and *S.* Indiana, with a widespread AMR spectrum is of concern. Moreover, several *Salmonella* isolates showed resistance to colistin, which is considered as one of the last-resort therapeutic options for the defense of multidrug-resistant Enterobacteriaceae (Elbediwi et al., 2019). This finding of colistin resistance agreed with results reported by other recent studies in *Salmonella* isolates from poultry and humans (Fortini et al., 2022; Tang et al., 2022). Of note, two carbapenems (imipenem and meropenem) resistant *Salmonella* isolates were also found in this study. Therefore, the spread of MDR *Salmonella* isolates, especially resistant to these clinically important antimicrobials, including fluoroquinolone, third-generation cephalosporins, colistin and carbapenems, will result in challenges with public health.

Phylogenetic analysis revealed a high level of genomic diversity of the *Salmonella* isolates collected from Qingdao region in China. Eight SNP clusters containing both chicken and human isolated collected in this study were identified through NCBI Pathogen Detection. Six of these clusters (PDS000043691.33, PDS000096798.86, PDS000106143.144, PDS000004748.67, PDS000101103.13, and PDS000026869.266) were featured by involving a large number of strains from different countries, indicating these isolates had been widely spread around the world. In contrast, the other two clusters (PDS000078427.2 and PDS000144678.1) formed distinct, locally restricted clades circulating in a limited number of cities in China. Most of the chicken and human isolates shared no common SNP clusters, indicating multiple origins of these isolates. Interestingly, the major SNP clusters found in the present study comprised numerous strains from clinical infections in China and other countries. This highlights the importance of conducting additional research on the dissemination of strains associated with these SNP clusters.

Mobile genetic elements (MGEs), such as bacteriophages and plasmids, are DNA segments that carry genes involving with DNA movement within genomes (Frost et al., 2005). The presence of intact Salmon 118,970 sal3, Gifsy-2, and Gifsy-1 was found at a higher frequency in the genomes of *S.* Typhimurium, *S*. I 4,[5],12:i:-, and *S.* Enteritidis, whereas such prophage types were seldom observed in other prevalent serotypes such as *S.* Indiana, *S*. Agona, and *S*. Derby. These findings indicate the presence of prophage types is associated with serotypes and may be related to the evolutionary history of different serotypes. Recently, a study of a large-scale investigation on *S.* Typhimurium has reported the common presence of the above-mentioned prophages in *S.* Typhimurium isolated in China (Wang et al., 2023). Furthermore, the specific phage, Salmon 118,970 sal3 was not only found in *Salmonella*, but also found in *E. coli* (Li et al., 2020) and *Morganella morganii* (Minnullina et al., 2019), suggesting that this phage has a relatively wide host range. The presence of Gifsy-1 and Gifsy-2 and their contribution to virulence in *S.* Typhimurium has been widely reported (Ho and Slauch, 2001).

Plasmids play a crucial role as the primary vehicles for horizontal gene transfer (HGT) in bacteria (De La Cruz and Davies, 2000). Among the isolates, the Col(pHAD28) plasmid replicon was the most common, and the IncF plasmid replicon was the second most common. Interestingly, the IncF is also one of the most prevalent plasmid replicons in *Salmonella* isolated from food animals in the United States (Mcmillan et al., 2019). The presence of Col(pHAD28) plasmid replicon was observed in a total of 14 serotypes identified in this study, indicating its high occurrence in *Salmonella* isolated from Qingdao region in China. Continuous monitoring of the spread of this plasmid is essential, as several studies have reported the presence of PMQR genes within this plasmid (Li et al., 2021; Hurtado et al., 2022).

Genomic analysis showed that the *Salmonella* isolates harbored different antimicrobial resistant genes. All of the studied *Salmonella* isolates harbored the *aac(6′)-Iaa* gene. However, *aac(6′)-Iaa* gene and similar genes usually are transcriptionally silent and rarely become transcriptionally active. AAC(6′)-Iaa is a typical member of the [A] family in that it acetylates tobramycin, kanamycin, and amikacin effectively but acetylates gentamicin ineffectively (Salipante and Hall, 2003). The mere presence of this gene does not confer aminoglycoside resistance in *Salmonella* (Magnet et al., 1999; Neuert et al., 2018). Besides, 81.4% (96/118) of the isolates resistant to the aminoglycoside class harbored different aminoglycoside resistance genes in addition to the *aac(6′)-Iaa* gene. Among diverse mechanisms of aminoglycoside resistance, enzymatic modification is the most prevalent mechanism in pathogenic bacteria, including *Salmonella* (Ramirez and Tolmasky, 2010; Biswas et al., 2019). Moreover, our study revealed 52/57 (91.2%) of the isolates resistant to the third-generation cephalosporins harbored different beta-lactam resistance genes, which were common detected in *S.* Enteritidis, *S.* Typhimurium, *S*. I 4,[5],12:i:-, and *S.* Indiana. The *bla* genes control the resistance to beta-lactam antimicrobials by hydrolyzing the beta-lactam ring, leading to antibiotic inactivation (Jacoby, 2009; Eguale et al., 2017). In this study, the frequently detected *bla*_TEM-1B_ gene (37.9%, 67/174) conferring resistance to ampicillin, is the dominant beta-lactam in most *Salmonella* serotypes worldwide (Eguale et al., 2017). Meanwhile, nine different CTX-M-type ESBL-producing were found in this study. During the last decade, the most frequently encountered (particularly in areas of Europe and Asia) ESBL genes were those encoding the CTX-M enzyme family, primarily carried by transferable plasmids and transposons (Cantón et al., 2012). The emergence of CTX-M-type ESBL-producing *Salmonella* has been reported in clinical cases, animals, and food samples worldwide, including China (Brown et al., 2018; Wang et al., 2020a). Five different *tet* resistance genes were found in 88/174 (50.6%) of the studied isolates, 84 of which were resistant to tetracyclines. Additionally, six plasmid-mediated quinolone resistance (PMQR) genes, with *qnrS1* prevalently detected, were identified in among the examined isolates belonging to different serotypes. These critical genes in *Salmonella* isolates from both chicken and human related samples present a tremendous public health concern. It is essential that the existence of the acquired antimicrobial resistant genes in bacterial genomes does not inevitably confer phenotypic resistance and vice versa.

Through NCBI’s Pathogen Detection platform, a total of 60 SNP clusters was categorized using isolates collected in this study (Supplementary Table S4). A SNP cluster is a cluster of isolates where each isolate is less than or equal to 50 SNPs distant from others, which offers higher resolution than serotype and MLST for outbreak surveillance purpose. In the designated SNP clusters, we identified several SNP clusters carrying PMQR genes. Some of these SNP clusters contained a large number of isolates from various isolation locations harboring specific PMQR genes, for example, *qnrS1* in PDS000112649.59 of *S.* Agona, *qnrB6* in PDS000102183.75 of *S.* Corvallis, and *qnrS1* in PDS000115660.28 of *S.* 4,[5],12:i:-; *qnr-*carrying strains with the same sequence types have been reported to be circulating globally with a high rate of quinolone resistance (Fernández et al., 2018; Zhang et al., 2020; Cadel-Six et al., 2021; Lee et al., 2021; Chen et al., 2023).

On the other hand, there are *qnr*-carrying isolates collected uniquely or mostly from China in SNP clusters with small numbers of matched isolates such as PDS000078519.2 of *S.* Derby, PDS000013413.32 of *S.* Thompson, and PDS000055747.16 of *S.* Typhimurium, indicating that these strains were circulating locally in specific regions in China and tend to be emerging threat to public health. Consistently, in a recent study investigating ciprofloxacin resistance in *Salmonella* from humans, food and animals collected in Shanghai, China, *qnr*-positive isolates were mostly *S.* Thompson, *S.* Derby and *S.* Typhimurium (Kuang et al., 2018a).

Apart from *qnr*-carrying isolates, this study also detected three *mcr*-carrying isolates. The isolate harboring *mcr-1.1* was found clustered in the SNP cluster PDS000042829.4 of *S.* I 4,[5],12:i:-, which contained a total of four *mcr-1.1*-carrying isolates uniquely from China, with one isolated from a diarrheal patient (Sun et al., 2023) and another one isolated from ready-to-eat pork (Wang et al., 2018). The two *mcr-9.1*-carrying isolates were designated to the SNP cluster PDS000101102.4 of *S.* Thompson. This SNP cluster had a total of 14 isolates collected from China, of which 11 were *mcr-9.1* positive and 9 were *qnrB4* positive. Interestingly, there are 8 isolates carrying both *mcr-9.1* and *qnrB4.* A recent study also reported a *S.* I 4,[5],12:i:- isolate carrying both *mcr-9.1* and *qnrA1* and it was found that these clinically relevant resistance genes were driven by IncHI2-ST1 plasmids (Vázquez et al., 2023).

Additionally, the *bla*_NDM-1_*-*carrying *S.* Enteritidis isolate was associated with the SNP cluster PDS000004748.67, which had a total of 258 matched isolates, but no other isolates within this cluster were *bla*_NDM-1_ positive, suggesting the *bla*_NDM-1_ positive isolate has recently acquired plasmid carrying *bla*_NDM-1_ gene. It should be paid great attention to as the NDM-1-harboring plasmids confer resistance to all β-lactams (Huang et al., 2017).

In summary, the frequently detection of *Salmonella* from retail chilled chicken carcasses in Qingdao, China, highlighted the importance of improving food hygiene measures to reduce the contamination and transmission of this bacterium. We found a considerable diversity of *Salmonella* serotypes and sequence types among both chicken and human, with MDR, posing a severe risk to food safety and public health. The finding of eight worldwide spread SNP clusters in this study emphasized additional research on the dissemination of strains associated with these SNP clusters. The MGE results showed high prevalence of plasmid replicons and prophages among *Salmonella* from both sources, and the presence of these MGEs associated with specific serotypes indicated different evolutionary history of these serotypes. Given the high prevalence of CTX-M type ESBL-producing and PMQR genes identified in this study, we strongly recommend that both clinical and veterinary sectors routinely test for AMR when resistance to these antimicrobials is detected in *Salmonella*. This will enhance monitoring and guide the selection of effective treatments. Overall, it is essential to continue monitoring the *Salmonella* serotypes, implement the necessary prevention and strategic control plans, and conduct an epidemiological surveillance system based on whole-genome sequencing.

## Data availability statement

The datasets presented in this study can be found in online repositories. The names of the repository/repositories and accession number(s) can be found in the article/Supplementary material.

## Author contributions

WW: Writing – original draft, Writing – review & editing. JC: Writing – original draft, Writing – review & editing. FLiu: Writing – review & editing. YH: Writing – review & editing. FLi: Writing – review & editing. XD: Writing – review & editing. YD: Writing – review & editing. SL: Writing – original draft, Writing – review & editing. JX: Writing – review & editing. ZZ: Writing – review & editing.

## Footnotes

^1^ https://card.mcmaster.ca/home

^2^ https://www.bioinformatics.babraham.ac.uk/projects/fastqc

^3^ https://github.com/tseemann/mlst

^4^ http://www.denglab.info/SeqSero2

^5^ https://github.com/tseemann/abricate

^6^ https://ngdc.cncb.ac.cn/gsa/
