# Supplementary material for: Genomic characterization of Salmonella isolated from retail chicken and humans with diarrhea in Qingdao, China
Source: Front Microbiol. 2023 Dec 18;14:1295769. doi: 10.3389/fmicb.2023.1295769 (PMC10757937; doi:10.3389/fmicb.2023.1295769)
Supplement: Supplementary file 2 [file Table_2.DOCX]

| Supplementary Table S2. Frequency of serovars among the studied isolates | | | | |
| --- | --- | --- | --- | --- |
| Serotypes | retail chilled chicken carcasses | | human with diarrhea | |
|  | Frequency | Ratio | Frequency | Ratio |
| Enteritidis | 15 | 17.0% | 20 | 23.3% |
| Indiana | 15 | 17.0% | 1 | 1.2% |
| Typhimurium | 15 | 17.0% | 15 | 17.4% |
| Derby | 11 | 12.5% | 4 | 4.7% |
| Agona | 6 | 6.8% | 7 | 8.1% |
| Thompson | 5 | 5.7% | 3 | 3.5% |
| Orion | 3 | 3.4% | - | - |
| Reading | 3 | 3.4% | - | - |
| Schwarzengrund | 3 | 3.4% | - | - |
| I 4,[5],12:i:- | 2 | 2.3% | 15 | 17.4% |
| Mbandaka | 2 | 2.3% | 3 | 3.5% |
| Newport | 2 | 2.3% | - | - |
| Corvallis | 1 | 1.1% | - | - |
| Give | 1 | 1.1% | 2 | 2.3% |
| I 4:b:- | 1 | 1.1% | - | - |
| Infantis | 1 | 1.1% | 1 | 1.2% |
| London | 1 | 1.1% | 6 | 7.0% |
| Rissen | 1 | 1.1% | 3 | 3.5% |
| Bareilly | - | - | 1 | 1.2% |
| Saintpaul | - | - | 2 | 2.3% |
| Stanley | - | - | 3 | 3.5% |

“-” means no detection.
