# Supplementary material for: Genomic characterization of Salmonella isolated from retail chicken and humans with diarrhea in Qingdao, China
Source: Front Microbiol. 2023 Dec 18;14:1295769. doi: 10.3389/fmicb.2023.1295769 (PMC10757937; doi:10.3389/fmicb.2023.1295769)
Supplement: Supplementary file 3 [file Table_3.DOCX]

| Supplementary Table S3. Frequency of sequence type | | | | | | |
| --- | --- | --- | --- | --- | --- | --- |
| ST | retail chilled chicken carcasses | | human with diarrhea | | Total | |
|  | Frequency | Ratio | Frequency | Ratio | Frequency | Ratio |
| 11 | 15 | 17.0% | 20 | 23.3% | 35 | 20.1% |
| 17 | 14 | 15.9% | 1 | 1.2% | 15 | 8.6% |
| 40 | 11 | 12.5% | 4 | 4.7% | 15 | 8.6% |
| 34 | 7 | 8.0% | 16 | 18.6% | 23 | 13.2% |
| 19 | 7 | 8.0% | 14 | 16.3% | 21 | 12.1% |
| 13 | 6 | 6.8% | 7 | 8.1% | 13 | 7.5% |
| 26 | 5 | 5.7% | 3 | 3.5% | 8 | 4.6% |
| 684 | 3 | 3.4% | - | - | 3 | 1.7% |
| 1628 | 3 | 3.4% | - | - | 3 | 1.7% |
| 413 | 2 | 2.3% | 3 | 3.5% | 5 | 2.9% |
| 45 | 2 | 2.3% | - | - | 2 | 1.1% |
| 241 | 2 | 2.3% | - | - | 2 | 1.1% |
| 155 | 1 | 1.1% | 6 | 7.0% | 7 | 4.0% |
| 516 | 1 | 1.1% | 2 | 2.3% | 3 | 1.7% |
| 32 | 1 | 1.1% | 1 | 1.2% | 2 | 1.1% |
| 36 | 1 | 1.1% | - | - | 1 | 0.6% |
| 42 | 1 | 1.1% | - | - | 1 | 0.6% |
| 96 | 1 | 1.1% | - | - | 1 | 0.6% |
| 99 | 1 | 1.1% | - | - | 1 | 0.6% |
| 1541 | 1 | 1.1% | - | - | 1 | 0.6% |
| 1836 | 1 | 1.1% | - | - | 1 | 0.6% |
| 3557 | 1 | 1.1% | - | - | 1 | 0.6% |
| 3558 | 1 | 1.1% | - | - | 1 | 0.6% |
| 29 | - | - | 3 | 3.5% | 3 | 1.7% |
| 469 | - | - | 3 | 3.5% | 3 | 1.7% |
| 50 | - | - | 2 | 2.3% | 2 | 1.1% |
| 909 | - | - | 1 | 1.2% | 1 | 0.6% |

“-” means no detection
