# Supplementary material for: Genomic characterization of Salmonella isolated from retail chicken and humans with diarrhea in Qingdao, China
Source: Front Microbiol. 2023 Dec 18;14:1295769. doi: 10.3389/fmicb.2023.1295769 (PMC10757937; doi:10.3389/fmicb.2023.1295769)
Supplement: Supplementary file 4 [file Table_4.DOCX]

Supplementary Table S4. Designated SNP clusters of *Salmonella* isolates

| Serotype | SNP cluster | Number of collected isolates | Number of isolates in SNP cluster | Source | ST | Average of plasmid replicon numbers | Average of intact prophage numbers | Average of ARG numbers | Average of resistance phenotype numbers |
| --- | --- | --- | --- | --- | --- | --- | --- | --- | --- |
| Agona | PDS000106143.144 | 6 | 2666 | human/chicken | 13 | 1.3 | 0.8 | 2.7 | 1.5 |
|  | PDS000112649.59 | 4 | 934 | human | 13 | 2.0 | 2.3 | 9.0 | 4.8 |
| Bareilly | PDS000011004.22 | 1 | 26 | human | 909 | 5.0 | 3.0 | 11.0 | 6.0 |
| Corvallis | PDS000102183.75 | 1 | 588 | chicken | 1541 | 3.0 | 2.0 | 10.0 | 6.0 |
| Derby | PDS000012191.29 | 1 | 101 | human | 40 | 1.0 | 3.0 | 12.0 | 4.0 |
|  | PDS000078487.2 | 1 | 9 | chicken | 40 | 3.0 | 1.0 | 20.0 | 6.0 |
|  | PDS000078519.2 | 1 | 19 | human | 40 | 4.0 | 2.0 | 16.0 | 6.0 |
|  | PDS000096058.5 | 2 | 51 | chicken | 40 | 3.5 | 3.0 | 3.0 | 1.0 |
|  | PDS000101114.3 | 1 | 17 | chicken | 40 | 1.0 | 3.0 | 5.0 | 1.0 |
|  | PDS000144672.1 | 2 | 2 | chicken | 40 | 2.0 | 0.5 | 6.0 | 4.0 |
|  | PDS000144677.1 | 3 | 3 | chicken | 40 | 2.0 | 1.0 | 7.3 | 3.3 |
|  | PDS000144679.1 | 2 | 3 | human | 40 | 3.5 | 1.0 | 9.5 | 8.0 |
| Enteritidis | PDS000004748.67 | 9 | 258 | human/chicken | 11 | 3.0 | 2.1 | 4.9 | 6.0 |
|  | PDS000026869.266 | 17 | 1562 | human/chicken | 11 | 3.6 | 2.4 | 5.5 | 4.1 |
|  | PDS000026888.108 | 2 | 242 | chicken | 11 | 2.5 | 2.0 | 1.0 | 1.0 |
|  | PDS000026902.16 | 2 | 26 | human | 11 | 3.0 | 3.0 | 1.0 | 4.0 |
|  | PDS000121788.43 | 1 | 3370 | chicken | 11 | 4.0 | 2.0 | 1.0 | 1.0 |
| Give | PDS000032733.16 | 2 | 44 | human | 516 | 3.0 | 2.0 | 8.0 | 1.5 |
| I 4,[5],12:i:- | PDS000042829.4 | 1 | 4 | chicken | 34 | 7.0 | 3.0 | 10.0 | 5.0 |
|  | PDS000096798.86 | 2 | 683 | human/chicken | 34 | 2.0 | 2.0 | 6.0 | 2.5 |
|  | PDS000115660.28 | 8 | 812 | human | 34 | 1.9 | 2.1 | 9.1 | 5.9 |
|  | PDS000140119.34 | 4 | 13298 | human | 34 | 2.3 | 2.0 | 7.5 | 6.5 |
|  | PDS000144683.1 | 2 | 2 | human | 34 | 6.0 | 2.0 | 15.5 | 6.0 |
| Indiana | PDS000043691.33 | 13 | 575 | human/chicken | 17/3558 | 4.5 | 0.5 | 14.2 | 7.0 |
|  | PDS000044150.2 | 1 | 40 | chicken | 17 | 1.0 | 1.0 | 20.0 | 8.0 |
| Infantis | PDS000043225.7 | 1 | 10 | chicken | 32 | 1.0 | 3.0 | 1.0 | 0.0 |
|  | PDS000064354.3 | 1 | 10 | human | 32 | 0.0 | 1.0 | 5.0 | 10.0 |
| London | PDS000003693.262 | 1 | 354 | human | 155 | 0.0 | 2.0 | 1.0 | 0.0 |
|  | PDS000078401.16 | 4 | 167 | human | 155 | 1.8 | 2.0 | 15.8 | 6.8 |
|  | PDS000078427.2 | 2 | 6 | human/chicken | 155 | 0.5 | 1.5 | 1.0 | 1.0 |
| Mbandaka | PDS000101127.2 | 1 | 6 | human | 413 | 1.0 | 1.0 | 1.0 | 1.0 |
|  | PDS000125082.33 | 1 | 1304 | chicken | 413 | 1.0 | 6.0 | 1.0 | 1.0 |
|  | PDS000144686.1 | 1 | 4 | human | 413 | 1.0 | 0.0 | 1.0 | 0.0 |
| Newport | PDS000042157.56 | 2 | 90 | chicken | 45 | 0.0 | 0.0 | 1.0 | 0.0 |
| Orion | PDS000049355.10 | 3 | 44 | chicken | 684 | 1.0 | 3.0 | 1.0 | 0.3 |
| Reading | PDS000144676.1 | 2 | 2 | chicken | 1628 | 1.5 | 5.0 | 5.0 | 1.0 |
| Rissen | PDS000001455.8 | 1 | 13 | chicken | 1836 | 1.0 | 2.0 | 1.0 | 3.0 |
|  | PDS000078410.5 | 1 | 24 | human | 469 | 2.0 | 3.0 | 8.0 | 3.0 |
|  | PDS000144684.1 | 1 | 3 | human | 469 | 3.0 | 3.0 | 2.0 | 2.0 |
| Saintpaul | PDS000144664.1 | 2 | 2 | human | 50 | 5.0 | 3.0 | 16.0 | 7.5 |
| Schwarzengrund | PDS000004036.89 | 1 | 157 | chicken | 96 | 0.0 | 5.0 | 1.0 | 2.0 |
|  | PDS000144681.1 | 2 | 11 | chicken | 241 | 1.5 | 0.5 | 6.0 | 3.5 |
| Stanley | PDS000144673.1 | 2 | 2 | human | 29 | 0.0 | 2.0 | 1.0 | 7.5 |
|  | PDS000144687.1 | 1 | 4 | human | 29 | 3.0 | 2.0 | 9.0 | 6.0 |
| Thompson | PDS000013413.32 | 2 | 85 | chicken | 26 | 1.0 | 2.5 | 14.0 | 7.0 |
|  | PDS000031681.25 | 1 | 72 | human | 26 | 0.0 | 0.0 | 1.0 | 7.0 |
|  | PDS000078414.6 | 1 | 7 | human | 26 | 4.0 | 2.0 | 11.0 | 8.0 |
|  | PDS000101102.4 | 2 | 14 | chicken | 26 | 4.0 | 4.0 | 11.0 | 6.5 |
|  | PDS000127939.4 | 1 | 10 | chicken | 26 | 5.0 | 2.0 | 19.0 | 9.0 |
|  | PDS000136184.2 | 1 | 3 | human | 26 | 1.0 | 1.0 | 2.0 | 6.0 |
| Typhimurium | PDS000026701.125 | 1 | 348 | chicken | 36 | 2.0 | 2.0 | 2.0 | 2.0 |
|  | PDS000042423.3 | 1 | 5 | chicken | 99 | 3.0 | 4.0 | 8.0 | 5.0 |
|  | PDS000043694.22 | 4 | 211 | chicken | 34 | 3.8 | 2.8 | 15.0 | 5.5 |
|  | PDS000055747.16 | 3 | 26 | human | 19 | 2.0 | 3.0 | 15.7 | 7.0 |
|  | PDS000078469.3 | 1 | 211 | chicken | 34 | 5.0 | 2.0 | 15.0 | 8.0 |
|  | PDS000101103.13 | 6 | 171 | human/chicken | 19 | 2.0 | 3.8 | 4.2 | 4.0 |
|  | PDS000106199.3 | 1 | 6 | human | 19 | 3.0 | 4.0 | 1.0 | 0.0 |
|  | PDS000144674.1 | 2 | 3 | chicken | 19 | 3.0 | 5.0 | 1.0 | 1.0 |
|  | PDS000144678.1 | 2 | 5 | human/chicken | 19 | 2.5 | 1.5 | 6.5 | 3.0 |
|  | PDS000144682.1 | 1 | 4 | chicken | 19 | 4.0 | 1.0 | 12.0 | 4.0 |
